# Next Generation Sequencing and Animal Models Reveal *SLC9A3R1* as a New Gene Involved in Human Age-Related Hearing Loss

**DOI:** 10.3389/fgene.2019.00142

**Published:** 2019-02-26

**Authors:** Giorgia Girotto, Anna Morgan, Navaneethakrishnan Krishnamoorthy, Massimiliano Cocca, Marco Brumat, Sissy Bassani, Martina La Bianca, Mariateresa Di Stazio, Paolo Gasparini

**Affiliations:** ^1^Department of Medicine, Surgery and Health Sciences, University of Trieste, Trieste, Italy; ^2^Institute for Maternal and Child Health – IRCCS “Burlo Garofolo”, Trieste, Italy; ^3^Sidra Medical and Research Center, Doha, Qatar; ^4^Heart Science Centre, National Heart and Lung Institute, Imperial College London, London, United Kingdom

**Keywords:** hearing loss, new gene discovery, zebrafish model, CRISPR-Cas9, next-generation sequencing

## Abstract

Age-related hearing loss (ARHL) is the most common sensory impairment in the elderly affecting millions of people worldwide. To shed light on the genetics of ARHL, a large cohort of 464 Italian patients has been deeply characterized at clinical and molecular level. In particular, 46 candidate genes, selected on the basis of genome-wide association studies (GWAS), animal models and literature updates, were analyzed by targeted re-sequencing. After filtering and prioritization steps, *SLC9A3R1* has been identified as a strong candidate and then validated by “*in vitro”* and *“in vivo”* studies. Briefly, a rare (MAF: 2.886e-5) missense variant c.539G > A, p.(R180Q) was detected in two unrelated male patients affected by ARHL characterized by a severe to profound high-frequency hearing loss. The variant, predicted as damaging, was not present in healthy matched controls. Protein modeling confirmed the pathogenic effect of p.(R180Q) variant on protein’s structure leading to a change in the total number of hydrogen bonds. *In situ* hybridization showed *slc9a3r1* expression in zebrafish inner ear. A zebrafish knock-in model, generated by CRISPR-Cas9 technology, revealed a reduced auditory response at all frequencies in *slc9a3r1*^R180Q/R180Q^ mutants compared to *slc9a3r1*^+/+^ and *slc9a3r1*^+/R180Q^ animals. Moreover, a significant reduction (5.8%) in the total volume of the saccular otolith (which is responsible for sound detection) was observed in *slc9a3r1*^R180Q/R180Q^ compared to *slc9a3r1*^+/+^ (*P* = 0.0014), while the utricular otolith, necessary for balance, was not affected in agreement with the human phenotype. Overall, these data strongly support the role of *SLC9A3R1* gene in the pathogenesis of ARHL opening new perspectives in terms of diagnosis, prevention and treatment.

## Introduction

Age-related Hearing loss (ARHL) is the predominant sensory impairment in the elderly (Huang and Tang, 2010; Kidd Iii and Bao, 2012), affecting millions of people worldwide (National Health and Nutrition Examination Survey [NHANES], 2015). Moreover, projections suggest that, in the next decade, the number of patients will double, largely due to an increased lifespan (Bowl and Dawson, 2015).

Age-related Hearing loss is characterized by bilateral and progressive hearing loss that usually starts at the high frequencies (Huang and Tang, 2010) causing communication difficulties associated with cognitive decline, social isolation and depression (Bowl and Dawson, 2015). It is a complex disease in which genetic and environmental risk factors (i.e., noise, smoking, alcohol, etc.) interplay (Bovo et al., 2011; Ohgami et al., 2013; Vuckovic et al., 2013, 2014). To date, it is still unclear whether ARHL is caused by (1) rare Mendelian gene variants with large effect size or (2) multiple variants each contributing to the disease. Despite significant research efforts carried out during the last decade, the genetics risk factors involved in ARHL are still mainly unknown. To date, only few ARHL susceptibility genes have been detected by genome-wide association studies (GWAS) (Friedman et al., 2009; Van Laer et al., 2010; Girotto et al., 2011, 2014; Wolber et al., 2014; Vuckovic et al., 2015), and thus there is a strong need to plan for new research activities aimed at understanding the molecular mechanisms underlying this diseases and to define possible targets for therapeutic and preventive plans.

The use of animal models is a powerful tool for the discovery and/or the validation of human disease-genes. In particular, zebrafish has become an attractive model for the study of the development and function of the vertebrate inner ear (Whitfield et al., 2002). Although the zebrafish ear does not contain an equivalent of the mammalian cochlea, many features (e.g., the organization and morphology of the supporting cells and hair cells, the mechanical stimulation of the ear, etc.) are conserved and analogous with other species (Haddon and Lewis, 1996; Nicolson, 2005; Abbas and Whitfield, 2009; Baxendale and Whitfield, 2016). Moreover, it has been shown that approximately 70% of human genes have, at least, one zebrafish ortholog (Howe et al., 2013) and a number of genes required for the hearing function in zebrafish have been also associated with auditory defects in mammals (i.e., mice and humans) further supporting their role across different species (Coimbra et al., 2002).

Starting from these considerations, we developed a combined strategy, based on: (1) Targeted Re-Sequencing (TRS) of a panel of 46 ARHL candidate genes (Morgan et al., 2018) in 464 patients, followed by (2) zebrafish models of the most interesting variants identified.

Here, we report the results obtained using this approach demonstrating the presence of a *SLC9A3R1* (also named Na^+^/H^+^ Exchange Regulatory Cofactor, *NHERF1*) pathogenic variant in two unrelated ARHL patients and finally that this variant has deleterious effects in a zebrafish knock-in (KI) model. These findings, together with a previous study demonstrating the involvement of *Slc9a3r1* in hearing loss in mouse (Kamiya et al., 2014), allow us to propose *SLC9A3R1* as an extremely promising new ARHL candidate gene.

## Materials and Methods

### Ethics Statement

#### Human

The study was reviewed and approved by the Ethics Committee of the Burlo Garofolo children’s hospital in Trieste (Italy) (2007 242/07). Written informed consent was obtained from each participant to the study and all the research was carried out according to the ethical standards defined by the Helsinki declaration.

#### Zebrafish

Zebrafish procedures were performed in accordance with Spanish and European laws, guidelines and policies for animal (Real Decreto 1201/05 (BOE 252, October 21, 2005 and European Directive 2010/63/EU on the protection of animals used for scientific purposes of October 20, 2010). This project was approved by the institutional Ethical Committee for Animal Experimentation of the PRBB, where ZeClinics conducts all experimental work (approval number CEA-OH/9421/2).

### Patients Recruitment

A large cohort of 464 ARHL patients coming from inbred (Friuli Venezia Giulia-FVG- Cohort, Carlantino Cohort) and outbred (Milan, Naples, Trieste) Italian populations was analyzed by TRS. The cohort consists of 258 males and 206 females, all aged over 50 and with high frequencies bilateral HL developed around the 5th decade of life [high-frequency pure tone average (PTAH) > 40dB]. No disorders in the external and middle ear, as well as any vestibular problem or syndromic features were present.

Furthermore, 350 healthy individuals (all aged ≥ 50 y.o with PTAH ≤ 25dB) coming from five out of six villages of the FVG cohort (Resia, Sauris, Clauzetto, San Martino del Carso, Illegio) were used as an internal control.

### Targeted Re-sequencing

A total number of 46 ARHL-candidate genes, including *SLC9A3R1*, were sequenced using Ion Torrent PGM^TM^ (Life-Technologies). Genes were selected according to data from (a) GWAS meta-analyses on isolated and outbred populations of European and Asian ancestry (Girotto et al., 2011, 2014; Wolber et al., 2014; Vuckovic et al., 2015); (b) literature updates and (c) animal models (Morgan et al., 2018).

Briefly, 10 ng of genomic DNA were used to construct DNA libraries using Ion AmpliSeq Library Kit 2.0 (Life Technologies). Template Ion Sphere Particles were prepared using Ion PGM Template OT2 200 kit and a single end 200 base-read sequencing run was carried out using Ion PGM sequencing 200 kit v2 (Life Technologies), on Ion Torrent PGM^TM^ (Life Technologies). Ten indexed patients’ libraries were sequenced simultaneously on each Ion 318 Chip. Sequencing data were then analyzed according to the Ion Torrent SuiteTM v3.6. The annotated SNVs∖INDELS were evaluated according to several *in silico* predictor tools (SIFT, Polyphen2, MutationTaster, LRT) (Ng and Henikoff, 2003; Chun and Fay, 2009; Schwarz et al., 2010; Adzhubei et al., 2013) and conservation across species (PhyloP) (Pollard et al., 2010). Variants were classified as ultra-rare (MAF < 0.001), rare (MAF < 0.01) or common (MAF > 0.01) based on the frequencies reported NCBI dbSNP build142^1^ as well as in 1000 Genomes Project^2^, NHLBI Exome Sequencing Project (ESP) Exome Variant Server^3^, ExAC Browser^4^ and gnomAD browser^5^. Finally, those variants most likely to be disease causing (i.e., rare and ultra-rare variants predicted as damaging by all *in silico* predictor tools) were analyzed by Sanger sequencing and tested in controls.

### SNP Genotyping

*SLC9A3R1* variant was checked in 350 healthy controls using a TaqMan SNP genotyping assay (Assay ID: C_164690872_10, Thermo Fisher Scientific). Reactions were performed according to the manufacturer’s instructions. Data were analyzed with the Taqman Genotyper Software (Thermo Fisher Scientific).

### Mutational Protein Modeling

The 3D structure of PDZ2 domain of the NHERF1 protein was retrieved from protein data bank [ID: 2KRG, (Bhattacharya et al., 2010)]. This structure was considered as wild type (WT) and used in discovery studio [DS, (Accelrys Inc., San Diego, CA, United States)] to produce a mutant model (R180Q) as previously described (Krishnamoorthy et al., 2011).

### Molecular Dynamics Simulations

The 3D structures of WT and mutant were used in the Groningen machine for chemical simulations (GROMACS) to perform molecular dynamics (MD) simulations. The atoms of the systems were prepared with GROMOS96 force field (van Gunsteren, 1996; Van Der Spoel et al., 2005; Hess et al., 2008). To solvate the protein, the SPC3 water model was used within a cubic box sized 1.5 nm (Berendsen et al., 1981). The counter ions were added to neutralize the systems and the periodic boundary conditions were applied in all directions. The prepared systems consist of 30202 and 30204 atoms in total. The LINCS (Hess et al., 1997) algorithm was used to constrain all the bond lengths and the SETTLE (Miyamoto and Kollman, 1992) algorithm was applied to constrain the geometry of the water molecules. A twin range cut-off was used: 0.8 nm for Van der Walls and 1.4 nm for electrostatic interactions, for managing long range interactions. The steepest descent algorithm was applied to energy minimize the systems with the tolerance of 2000 Kj/mol/nm. Subsequently, these structures were pre-equilibrated with 100 ps simulation before performing the production MD simulation for 25 ns with a time-step of 2 fs at constant temperature (300 K), pressure (1 atm) and number of particles, without any position restraints (Berendsen et al., 1984). The structures were collected at regular interval for every 100 ps to trace the trajectories and the tools in PyMOL ^6^, DS and GROMACS were utilized for analyzing the structures and their molecular interactions.

### Cluster Analysis and Surface Map

To represent the structure from the MD simulations, each trajectory with 2500 structures were classified into clusters based on their structural deviations. A structure from the top ranked cluster was chosen for representation as it was frequently occurring conformation. The representative structures were further used in DS for mapping the surface and to analyze the modifications on the binding surface (potential sites for binding partners).

### *In vitro* Molecular Cloning

The impact of the identified mutation on mRNA and protein levels was tested by transient transfection in HEK 293 cells using expression clones containing either the wild-type (WT) or the mutant cDNA. cDNAs were cloned into a pCMV6-Entry vector (Origene, Rockville, MD, United States), Myc-tagged.

The calcium phosphate transfection method was used (Kingston et al., 2003). Forty-eight hours after transfection total cell proteins and RNAs were prepared and analyzed by Western Blot (WB) and quantitative Real Time PCR (qRT-PCR), respectively.

### Western Blot Analysis

For protein analysis, HEK 293 cells were lysed in IPLS buffer (50 mM Tris-HCL pH7.5, 120 mM NaCl, 0.5 mM EDTA and 0.5% Nonidet P-40) supplemented with proteases inhibitors (Roche). After sonication and pre-clearing, protein lysate concentration was determined by Bradford Assay (Bio-Rad). An 8% polyacrylamide gel was used for protein electrophoresis. After blotting, membranes were blocked with 5% skim milk in Tris-buffered saline, 0.1% Tween 20 (TBST) and then incubated with primary c-Myc Antibody 9E10 monoclonal (Santa Cruz) overnight. Secondary antibodies [anti-mouse antibody (Santa Cruz)] were diluted in blocking buffer and incubated with the membranes for 45 min at room temperature. Proteins were detected with the ECL detection kit (GE Health Care Bio-Sciences).

Housekeeping proteins (e.g., β-actin or Hsp90) were used as an internal control for protein loading as well as for reference in the WB analysis.

### Quantitative Real-Time PCR (qRT-PCR)

RNA was extracted from cell pellets using High Pure RNA isolation Kit (Roche). Total RNA (1 μg) was reverse transcribed to cDNA using Transcriptor First Strand cDNA Synthesis kit (Roche). qRT-PCR was performed using standard PCR conditions in a 7900HT Fast Real Time PCR System (Applied Biosystems) (i.e., 95°C for 10′, 40 cycles of 95°C for 15″ and 60°C for 1′, followed by a dissociation stage of 95°C for 15″, 60°C for 15″ and 95°C for 15″) with Power SYBR Green PCR Master Mix (Thermo Fisher Scientific). Gene-specific primers were designed by using Primer3Web software^7^. All experiments were performed in biological triplicate. Expression levels have been standardized to Neo gene expression and all data have been analyzed using the 2^−ΔΔCT^ Livak Method (Livak and Schmittgen, 2001).

### Zebrafish Husbandry

Zebrafish KI lines used in this study were generated, analyzed phenotypically and maintained at ZeClinics (Barcelona, Spain). Two different zebrafish transgenic lines have been used as a background for the KI lines generation:

(1) Tg(brn3c:mGFP), which is a stable reporter line where a GFP tagged to the membrane is expressed under the control of the brn3c promoter (Xiao et al., 2005), thus specifically labeling a subset of retinal ganglion cells, hair cells of the inner ear and the lateral line neuromasts (Di Donato et al., 2013).
(2) Tg(isl3:GFP), which is a stable reporter line where a GFP is expressed in the cytoplasm of sensory neurons under the control of the isl3 promoter (Pittman et al., 2008).

Embryos were maintained in petri dishes at 28.5 Celsius. Developmental stages were evaluated as hours and days post-fertilization (hpf and dpf). All zebrafish experimental protocols were approved by the Generalitat de Cataluña.

### Gene Expression in Zebrafish Larvae

Gene expression in Zebrafish larvae (5 dpf) was performed by whole mount *in situ* hybridization (ISH). At this stage, inner ear is developed to allow functional hearing, which implies hair cells involved in that function are mature. Synthesis of antisense RNA and whole-mount *in situ* hybridization were performed as previously described (Thisse et al., 2004).

Specific riboprobes intended to recognize *slc9a3r1* mRNA were designed (Slc9a3r1_Fw:ATGTCCAGCGACCTCAGGCC; Slc9a3r1_Rv_T7: TAATACGACTCACTATAGGGCTCCAGTCC ATCTGCGGAGCTC).

cDNAs were amplified by PCR, using Expand High Fidelity PCR System (Roche), from a custom Zebrafish cDNAs library obtained by RT-PCR from (SuperScript III Rev Transcript kit, Invitrogen) a mRNA pool coming from 5 dpf Zebrafish larvae [Trizol mRNA extraction protocol, Trizol (Sigma-Aldrich)]. A T7 sequence linker in reverse primers was included to directly use the synthesized PCR products as templates to amplify the reverse digoxigenin-labeled riboprobe to be used for ISH.

Once dissected, embryos were fixed in 4% paraformaldehyde (PFA) over night (O/N), and then dehydrate with increasing concentrations of Metanol 0,1% Tween20 in PBS (PBT) for long-term storage (25, 50, and 75%). Embryos were then rehydrated and treated with proteinase K (Sigma-Aldrich). Afterwards embryos were incubated with a hybridization mix [i.e., 50% Formamide, 5× saline-sodium citrate (SSC), 0.1% Tween 20, Citric acid to adjust HM to pH 6.0 (460 μl of 1 M citric acid for 50 ml of mix), 50 μg/ml heparin, 500 μg/ml tRNA] containing the riboprobe (dilution: 1:100) and then with antibody against digoxigenin [Peroxidase-conjugated anti-DIG Fab (Roche), dilution 1:1000]. Nitroblue Tetrazolium (NBT: 3,5 μl of 50 mg/ml NBT solution in 1 ml of alkaline Tris buffer) was used with the alkaline phosphatase substrate 5-Bromo- 4- Chloro-3-Indolyl Phosphate (BCIP: 4,5 μl of 50 mg/ml BCIP solution in 1 ml of alkaline Tris buffer).

In order to check gene expression in hair cells, marked with GFP through its activation by the Brn3c promoter, secondary antibody against GFP was used (rabbit anti-GFP, Torrey Pinnes; 1:400).

Stained embryos were processed for imaging through 2 different methods:

(1) Whole embryos were imaged with a bright field stereoscope to determine the overall expression pattern.
(2) Transversal sections were acquired though a cryostat (20 μm width) to determine precisely the mRNA location within the inner ear across three different anterioposterior positions. Gene expression pattern was compared with the position of hair cells, marked with GFP through its activation by the Brn3c promoter. Sections were imaged with Leica DM5 light/fluorescent microscope at 40× magnification.

### Generation of *slc9a3r1* Knock-In (KI) Zebrafish Line

To generate the KI, we designed a strategy where double-strand breaks (DSBs), generated by CRISPR technology, are repaired through homologous recombination (HR) DNA repair mechanism (Cong et al., 2013). HR has been enhanced through the co-injection of Rad51 protein (Takayama et al., 2017) and providing a single-stranded oligonucleotide (ssOligo) that contains two homologous arms flanking the desired mutation.

### Synthesis of *slc9a3r1* sgRNA

After assessing Slc9a3r1 protein conservation with its human orthologous, single guide RNAs (sgRNAs) were synthesized *in vitro* from double strand DNA that contained a T7 promoter region, a specific CRISPR recognition site (20N spacer) and a guide sequence that allows recognition by CAS9. This double strand DNA was synthesized by the partial annealing of a constant oligo (80 bps) and a gene-specific oligo (60 bps). After annealing, DNA was fill-in by T4 polymerase (Gene-specific oligo: TAATACGACTCACTATA-N20-GTTTTAGAGCTAGAAATAGCAAG; Constant oligo: AAAAGCACCGACTCGGTGCCACTTTTTCAAGTTGATAACGGACTAGCCTTATTTTAACTTGCTATTTCTAGCTCTAAAAC).

### CRISPR Injection and Screening

**FIGURE 1:**
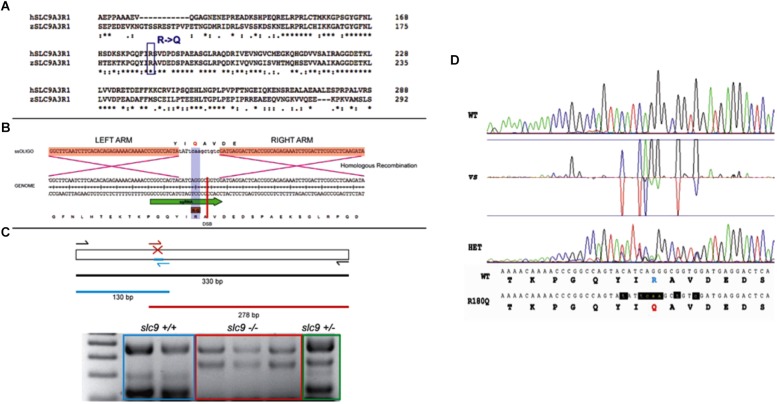
CRISPR-Cas9 strategy for the generation of the R180Q-*slc9a3r1* K/I model. **(A)** Comparison of human and zebrafish SLC9A3R1 amino acid sequence. Purple rectangle outlines R180 conservation among human and zebrafish. **(B)** Scheme displaying the nucleotide and amino acid translated sequence present at the target genome (below) and the ssOligo (above) used for modifying the target sequence. Below, green arrow in genome sequence outlines the sgRNA site. Above, red squares in ssOligo outline homologous recombination right and left arms. **(C)** Scheme displaying the PCR strategy used to identify potential mutants. **(D)** Example of R180Q-Slc9a3r1 mutant identified through the comparison of Sanger sequences obtained from a wild type (above) and a heterozygous individual (below). Both sequences display nucleotide and translated amino acid sequence. Above, targeted amino acid R180 is displayed in green. Below, modified amin oacid Q180 is displayed in red and modified nucleotides are displayed in red and lower case.

Tg(brn3c:mGFP)or Tg(isl3:GFP) embryos were injected with a mix of sgRNA, Cas9 (mRNA), ssOligo and Rad51 human Protein. 20–25 embryos of 48 hpf were selected and pooled for genomic DNA extraction. Genotyping was performed using specific diagnostic primers, a pair of primers to amplify the surrounding area (SLC9A3R1_Fw/SLC9A3R1_Rv = 379 bps; SLC9A3R1_Fw: CCCTTTGTAGGACTCGAAGAACGAG; SLC 9A3R1_Rv: TGTTCCAAACTAAGCCAGAGCAGAAC) and one primer to discriminate the mutated region (SLC9A 3R1_KI_Fw/SLC9A3R1_Rv = 278 bps; SLC9A 3R1_KI_Fw: CTTCTTGCGCAAATGGTTGTTG) (Figure 1C).

The remaining injected animals were grown until sexual maturity. F1 animals were Sanger sequenced to identify positive targeted mutants generated through CRISPR KI. These animals were incrossed to obtain F2 homozygous larvae to perform their phenotypical analysis.

### Genotyping

Genomic DNA of single larvae from the different experiment was extracted using Extract-N-AmpTM Tissue PCR Kit (Sigma). For the *Slc9a3r1* KI a first PCR reaction was performed using the primers SLC9A3R1_Fw and SLC9A3R1_Rv and the following conditions: 94°C 2 min, 25× (94°C 15 s, 58°C 30 s, 72°C 1:10 min), 72°C 7 min, 4°C hold. Then, a nested PCR reaction was performed using the primers SLC9A3R1_Fw, SLC9A3R1_Rv, SLC9A3R1_INT_MUT_Fw (5′-CTCATCCACCGCCCTGATG-3′), SLC9A3R1_INT_WT_Rv (5′-CCGGCCAGTATATTCAAGCTGTC- 3′) and the same thermal protocol.

### *slc9a3r1* KI Sound Response

Six day-old larvae obtained by pairwise mating of adult tg(brn3c:mGFP;R180Q- *slc9a3r1^+/R180Q^*) were tested to determine their ability to respond to sound stimuli at different frequencies, measuring the acoustic startle response (Bhandiwad et al., 2013). The EthoVision XT 12 software and the DanioVision device from Noldus Information Technologies (Wageningen, Netherlands) were used. This closed system consists of a camera placed above a chamber with circulating water and a temperature sensor that is set at 28°C. A 96-wells plate is placed in the chamber, which can provide different stimuli (light/dark environment, tapping, sound) controlled by the software. For each sound frequency tested, larvae were left for 10 min in dark for acclimation, 2 min with the light on (natural locomotor behavior of zebrafish is active in dark and immobile in light) and then the sound stimulus was provided. Finally, a tapping was given to the 96-wells plate in order to verify the absence of general locomotor defects. The system provides tapping stimuli that range from 1 to 8 arbitrary units (AU). An intensity of 3 AU was chosen for the experiments (1 and 2 were too weak to induce any response in normal larvae and higher intensities could over stimulate larvae and mask possible subtle effects).

The final readout to assess the capacity to respond to sound was the percentage of stimuli responding 6 day-old larvae.

Four different sound stimuli (300, 325, 350, and 375 Hz) that showed the highest percentage of responding larvae were tested.

Then genomic DNA was extracted from single larvae to identify their specific genotype.

To further verify the normal locomotion activity of *slc9a3r1^R180Q/R180Q^* larvae, we analyze their movement during the entire trial and compare it with the wild-type.

### Hair Cell Characterization

#### Hair Cell Number

In order to test the number of functional hair cells in the saccular macula (the sensory patch responsible for hearing) tg(brn3c:mGFP;R180Q- *slc9a3r1^+/R180Q^*) adult carrier were incrossed and 6 dpf progeny were intra-ear injected with FM1-43FX, a vital dye that penetrates in only mature and fully functional hair cells through calcium channels. In particular, 6 dpf larvae were anesthetized using Tricaine (20 μM) and 1 nl of FM1-43FX (stock solution 300 μM) was microinjected into the inner ear lumen (intra-ear). Once injected, larvae were fixed with 4% PFA 2 h at room temperature and then incubated with PBS-Triton 2% O/N, in order to dissolve the otoliths. The larvae were then embedded on their lateral sides in 1% low melting point agarose and the hair cells expressing membrane GFP (mGFP) and that internalized the FM1-43X were imaged using a SP8 Leica confocal microscope and *Z*-stacks spanning the entire saccular macula were taken (one *z*-plane imaged every 1 μm). Raw data was analyzed with FIJI software (Schindelin et al., 2012).

Confocal imaging of the saccular macula of *slc9a3r1*^+/+^, *slc9a3r1*^+/R180Q^ and *slc9a3r1*^R180Q/R180Q^ was done and images where analyzed. An in-house FIJI- based macro allows us to 3D reconstruct the entire saccular macula, measure the GFP (brn3c) and DsRed (FM1-43FX) signals and estimate the total number and the number of mature hair cells.

#### Hair Cells Morphology and Polarity

To test sensory hair cells morphology and orientation, 6 dpf larvae from tg(brn3c:mGFP;R180Q-*slc9a3r1*^+/R180Q^) adult pairwise mating were cryosectioned and an immunohistochemistry against the acetylated-tubulin, which labels the kinocilium, was performed. This staining, together with the membrane GFP signal help to detect alteration in hair cell morphology or in the orientation of the hair cell bundles.

Six dpf larvae were fix with 4% PFA 2 h at room temperature and then incubated with PBS-Triton 2% O/N in order to dissolve the otoliths. After several washes with 0.1%-Tween-20 PBS, larvae heads were incubated 1 h in 15% sucrose (in PBS) then in 15% sucrose/7.5% gelatin and placed in cryomold in the desired orientation (transversally for hair cell morphology, laterally for hair cell bundle orientation), while tails were used for genotyping. Blocks were frozen in 2-Methylbutane for tissue preservation and cryosectioned at 30 μm on a Leica CM 1510-1 cryostat. Sections were collected on Superfrost slides. Then the inner ear containing slides were blocked with blocking solution [0.1%Tween-20 in PBS (PBT), 2% bovine serum albumin (BSA) and 10% goat serum] for 1.5 h at RT. Mouse anti-acetylated-tubulin (Sigma; 1:1000) and rabbit anti-GFP (Torrey Pines; 1:400) were incubated overnight at 4°C in blocking solution. After washing with PBT for the whole day, anti-rabbit Alexa488 and anti-mouse Alexa648 (Invitrogen; 1:400) were incubated overnight at 4°C in blocking solution. Slides were then mounted with mowiol and imaged using a SP8 Leica confocal microscope and *Z*-stacks spanning the entire saccular macula were taken (one *z*-plane imaged every 1 μm). Raw data were analyzed with FIJI software in order to generate 3D reconstruction of the saccular macula or analyze the hair cell bundle orientation. To quantify and generate the graphs showing the ciliary orientation a Python-based in-house program was used.

For every single cell, we determine the orientation based on the position of the kinocilia and plot every cell on an Angle’s plot (Inoue et al., 2013) based on their polarization and finally generate a Polar plot to represent the percentage of the cells oriented in the dorsal, ventral, anterior, and posterior direction.

### Stato-Acoustic Ganglion Characterization

The number of neurons in the posterior stato-acoustic ganglion (SAG) (which innervate the saccular macula) was tested.

Adults Tg(isl3:mGFP;R180Q-*slc9a3r1*^+/R180Q^) were incrossed to obtain progeny. Six dpf larvae were fixed with 4% PFA 2 h at room temperature and then incubated with PBS-Triton 2% O/N in order to dissolve the otoliths. The larvae were then embedded on their sides in 1% low melting point agarose and the sensory neurons expressing GFP were imaged using a SP8 Leica confocal microscope and *Z*-stacks spanning the entire posterior SAG were taken (one *z*-plane imaged every 1 μm). Raw data were analyzed with FIJI software (Schindelin et al., 2012).

Using a FIJI-based in-house macro the entire posterior SAG was 3D reconstructed the number of the sensory neurons was calculated.

### Otolith Analysis

To test the size of the otolith, a crystalline structure contacted by the hair cell ciliary array and essential to drive the movement of the hair cell bundle, we incrossed tg(brn3c:mGFP;R180Q-*slc9a3r1*^+/R180Q^) adult carriers and 6 dpf progeny was anesthetized using Tricaine (20 μM), placed on their side, and imaged with Leica M165 Stereoscope before genotyping.

The images were analyzed with FIJI software and the area of the posterior otolith was measured.

### Statistical Analysis

Behavioral and otolith differences between *slc9a3r1*^+/+^, *slc9a3r1*^+/R180Q^, and *slc9a3r1*^R180Q/R180Q^ were evaluated with an unpaired *T*-test with Welch’s correction.

The significance threshold was set at 0.05.

## Results

**FIGURE 2:**
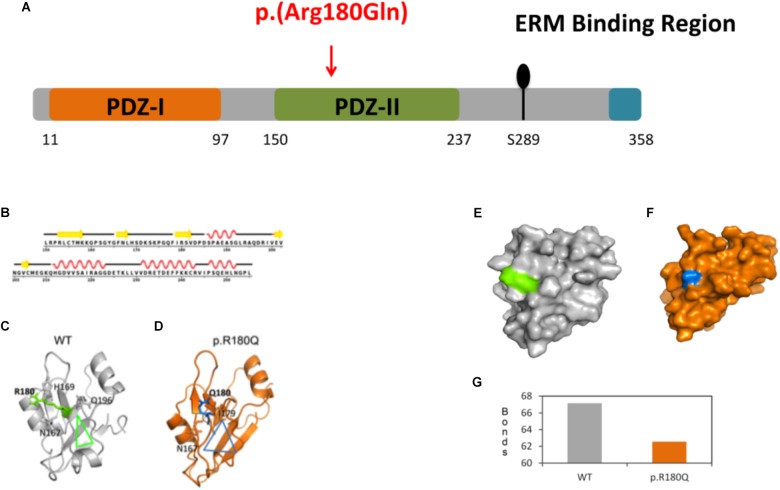
Molecular dynamics (MD) simulations and conformational analysis of NHERF1. **(A)** Schematic representation of NHERF1 protein domains. **(B)** Secondary structural details of the PDZ-II domain. **(C,D)** Secondary structural elements of the representative structures of the WT and mutated proteins. **(E,F)** Modification of the entire domain’s structure. **(G)** Representation of the total number of hydrogen bonds of the WT residue compared to the mutated one [from 67 (WT) to 62 (p.(R180Q)].

A total of 464 ARHL Italian patients were screened with a TRS panel of 46 ARHL candidate genes (Morgan et al., 2018). A mean of 33.5 megabases of raw sequence data were available for each subject. The coverage, on 95% of the targeted region, was at least 20-folds, with a 270-fold mean-depth total coverage. On average 333 single nucleotide variants (SNVs) and small insertions/deletions (INDELs) were called for each patient. After applying the filtering procedure described in the Section “Materials and Methods,” a missense variant at the heterozygous state, c.539G > A, p.(R180Q) in *SLC9A3R1* (NM_004252.4) gene was detected in two unrelated ARHL male patients (582130, aged 79, and 593486, aged 63). It affects the PDZ2 domain of NHERF1 protein (Figure 2A) and all *in silico* predictor tools classified it as damaging (Supplementary Figure S1B). Both subjects come from the same isolated village located in North Eastern Italy (Friuli Venezia Giulia-FVG cohort) and they were recruited thanks to a huge project on isolated community in Italy (INGI consortium) based on a volunteer study. Both patients show severe to profound high-frequency hearing loss resembling a sensory ARHL phenotype and the age of onset was 48 y.o. (582130) and 51 y.o. (593486) (Supplementary Figures S1A,B) without vestibular signs or symptoms. Additional clinical data include: (1) patient 582130: normal BMI, high pressure treated with drugs. During his life he was affected by Tuberculosis (TBC), lung cancer and dislipidemia. Habits: no smoking and limited alcohol consumption; (2) patient 5934486: overweight, high pressure treated with drugs, asthma treated with drugs, sarcoidosis. Habits: no smoking and no alcohol consumption. No other clinical features were detected in the patients nor exposure to noise.

The c.539G > A, p.(R180Q) variant has a frequency of 0.002 in our cohort of 464 ARHL patients and it is described in gnomAD browser with a minor allele frequency (MAF) of 2.886e-5 (rs146832150) (date of access 21/08/2018).

We confirmed the absence of the variant in all the available relatives of the patients (2 healthy daughters of 582130) as well as any other ARHL patient and control from the same small community (15 ARHL cases and 37 healthy controls). Moreover, 350 additional controls coming from the other five villages of the FVG cohort have been tested, confirming the absence of the *SLC9A3R1* variant.

### Protein Simulations and Conformational Analysis

Protein modeling and molecular dynamics (MD) simulations were used to test the impact of the mutation at molecular level. The secondary structural details of the PDZ2 domain (Figure 2B) show that residue R180 is located in the surface of a well-structured β sheet (Figure 2C) underlying its importance for the protein structure and binding activity. MD simulations revealed that secondary structural elements of both the wild-type (WT) and the mutant are stable during simulations. However, a major change was observed in the side chain of Q180 compared to R180, which further affects their hydrogen bonding partners (R180: H169, Q196 and N167 and Q180: I179 and N167) in the nearby region (Figure 2C,D). The outcome is a series of local network changes leading to rearrangements of neighboring loops of the mutational spot (Figure 2C,D, see the triangles for the impact) and following modifications on the surface of the domain itself whose entire conformation is significantly modified (Figure 2E,F). This finding correlates with the change in the total number of hydrogen bonds from 67 (WT) to 62 (p.(R180Q)) (Figure 2G). Overall, the modeling suggests a significant conformational change that can alter the normal function of the domain and the protein itself.

### *In vitro* Experiments: qRT-PCR, Western Blot, Immunostaining

In order to understand the effect of the mutated allele on RNA and protein stability, HEK 293 cells have been transfected with expression vectors containing either the WT or the mutant cDNA. qRT-PCR and WB analysis 48 h after the transient transfection did not reveal any difference in the levels of both mRNA and protein between WT and mutant (Supplementary Figure S2).

Moreover, immunostaining assay on transfected HeLa cells revealed no differences in the cellular localization of the mutated protein compared to the WT (Supplementary Figure S2).

### *In vivo* Experiments: Zebrafish KI Model

#### *In situ* Hybridization

**FIGURE 3:**
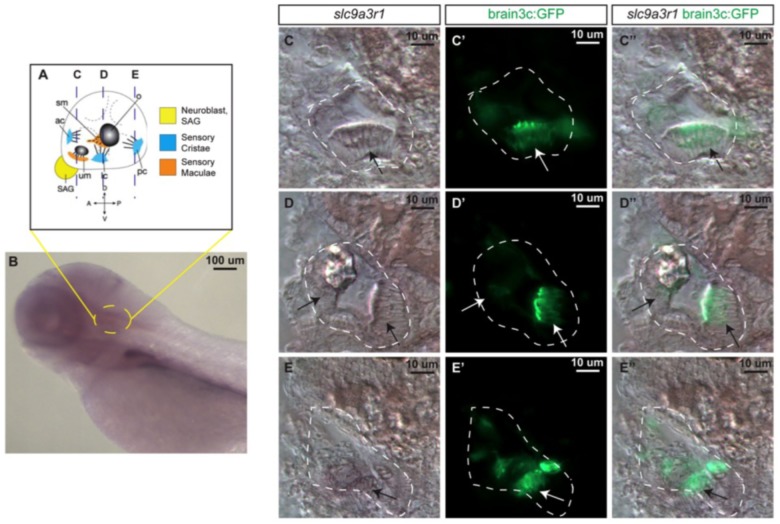
*slc9a3r1* expression analysis by *in situ* hybridization (ISH). **(A)** Schematic of 5 dpf inner ear cellular organization. The position of transversal views **(C–E)** is outlined by dotted lines. **(B)** Lateral view of the anterior region of the 5 dpf larvae. Yellow circle delimits the inner ear location. **(C,C’,C”)** Transversal view of the anterior region of the inner ear. The inner ear is outlined by a white dotted line. Arrows point to hair cell patches location. **(C)** *slc9a3r1* ISH. **(C’)** brain3c:GFP. **(C”)** *slc9a3r1* and brain3c:GFP merged image. **(D,D’,D”)** Transversal view of the medial region of the inner ear. The inner ear is outlined by a white dotted line. Arrows point to hair cell patches location. **(D)** *slc9a3r1* ISH. **(D’)** brain3c:GFP. **(D”)** *slc9a3r1* and brain3c:GFP merged image. **(E,E’,E”)** Transversal view of the posterior region of the inner ear. The inner ear is outlined by a white dotted line. Arrows point to hair cell patches location. **(E)** *slc9a3r1* ISH. **(E’)** brain3c:GFP. **(E”)** *slc9a3r1* and brain3c:GFP merged image.

Prior to the generation of the KI zebrafish model, *slc9a3r1* expression was tested in 5 dpf zebrafish larvae, by whole mount *in situ* hybridization (Figure 3). *slc9a3r1* mRNA was detected broadly across the larvae, but its expression was enriched in the hematopoietic lineage, liver primordium (Figure 3B) and in the inner ear (Figure 3C–E).

#### KI Hearing Phenotype

**FIGURE 4:**
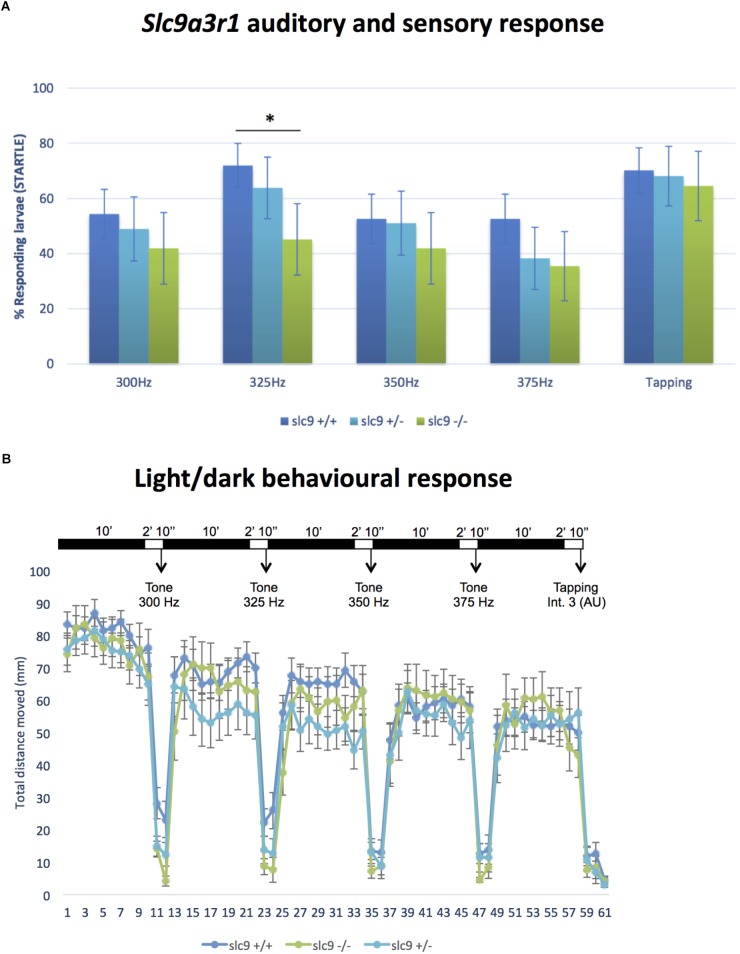
Auditory/sensory response and light/dark behavioral response in Zebrafish larvae. **(A)** Response of *slc9a3r1* KI animals compared to the wild-type ones to all the different sound stimuli tested (number of tested animals: *slc9a3r1*^+/+^ *N* = 57, *slc9a3r1*^+/-^ *N* = 47, *slc9a3r1*^R180Q/R180Q^ *N* = 31). Statistically significant difference indicated with “*” (^∗^*p*-value < 0.05). **(B)** Behavior and response activity to visual stimuli of *slc9a3r1*^R180Q/R180Q^ compared to *slc9a3r1*^+/+^ and *slc9a3r1*^+/-^. Tapping intensity is indicated as arbitrary unit (AU).

CRISPR/Cas9 technology has been used to generate the KI model (R180Q- *slc9a3r1*), in which has been introduced a precise nucleotide modification mimicking the human mutation (Figure 1A–D). Overall 135 six days post fertilization (dpf) larvae obtained by pairwise mating of adult tg (brn3c:mGFP;R180Q-*slc9a3r1*^+/R180Q^), were tested to determine their capacity to respond to sound stimuli at different frequencies (300, 325, 350, and 375). In general, for all stimuli, the response of *slc9a3r*1^R180Q/R180Q^ KI larvae (*n* = 31) was lower as compared to that of heterozygous *slc9a3r1*^+/R180Q^ (*n* = 47) and WT animals (*n* = 57). Interestingly, a statistical significant difference in sound perception at 325 Hz was definitely evident between the WT and the homozygous mutants (*P* = 0.0132), and a worsening hearing perception was also noticed in the heterozygous animals (Figure 4A) while no variation in the ability to respond to mechanical stimuli was observed in comparison to the wild type siblings.

#### Behavioural Test

Locomotion was studied after light/darkness stimuli to verify that response differences to sound stimuli among WT and mutant siblings were not promoted by general defects in locomotive behavior.

Wild type Zebrafish larvae show a relatively high swimming activity in darkness when compared to the activity in light (de Esch et al., 2012). No differences have been observed for the *slc9a3r1*^+/R180Q^ and *slc9a3r1*^R180Q/R180Q^ models (see Figure 4B). The graph shows normal behavior and response to visual stimuli of homozygous *slc9a3r1* mutants compared to heterozygous and WT, demonstrating that the impairment observed in sound response is specific to hearing function and not to defects in central nervous system.

To further investigate the role of this gene/mutation, several functional tests in the KI model have been performed and, among them, a significant reduced otolith size has been detected.

#### Measurement of the Otolith Size

Despite fish vestibular-acoustic system presents several functional elements that are very similar to the human ones (e.g., hair cells, supporting cells, etc.), they do not have a counterpart of the cochlea. Moreover, in fish inner ear the otoliths are essential structures for balance and hearing. Otoliths are crystalline structure contacted by the hair cell ciliary array and are essential to drive the movement of the hair cells bundle and start the electrical signaling in case of sound (Riley and Phillips, 2003). The alteration of the size, shape or the absence of this structure could have striking effects on the hearing ability (Inoue et al., 2013). For this reason, we looked for any difference in the otoliths of WT compared to that of the mutated animals.

**FIGURE 5:**
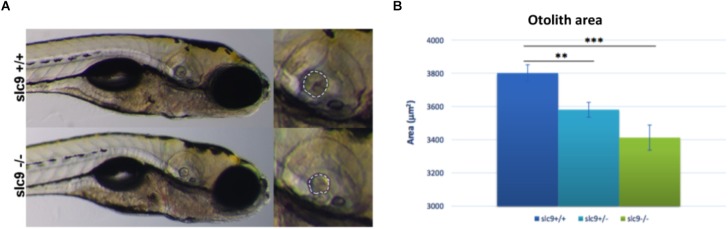
Measurement of the otolith size. **(A)** Measurement of the posterior otolith in *slc9a3r1*^+/+^ and *slc9a3r1*^R180Q/R180Q^ larvae. **(B)** Measurement of posterior otolith area of *slc9ar1* larvae (number of tested animals: *slc9a3r1*^+/+^ *N* = 57, *slc9a3r1*^+/-^ *N* = 70, *slc9a3r1*^R180Q/R180Q^ *N* = 32). ^∗∗^*p*-value < 0.005 and ^∗∗∗^*p*-value < 0.0005.

Results shows a 5,8% reduction of the otolith size in *slc9a3r1*^+/R180Q^ compared to WT (*P* = 0.0014) and an even more increased 10.2% reduction in the size of the otolith of *slc9a3r1*^R180Q/R180Q^ compare to WT (*P* < 0.0001) (Figure 5A,B). These results thus explain the hearing impairment observed in the *slc9a3r1*^R180Q/R180Q^ KI larvae.

Other functional tests demonstrated no alterations in the hair cells number and morphology as well as in the count of neurons, as reported below.

#### Hair Cells Number and Morphology

**FIGURE 6:**
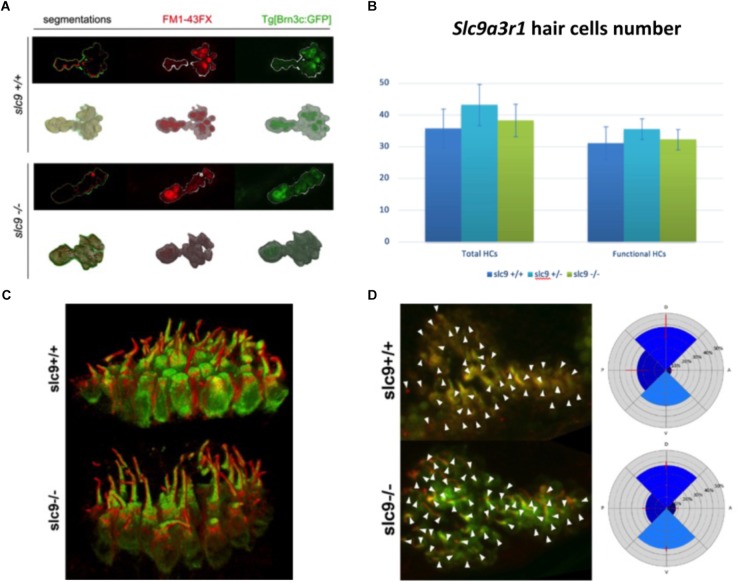
Hair cells number, morphology and orientation in Zebrafish larvae. **(A)** 3D reconstruction of the saccular macula. Hair cells are marked with GFP. FM1-43FX dye labels only mature and fully functional hair cells. **(B)** Saccular hair cells count in *slc9a3r1* larvae (number of tested animals: *slc9a3r1*^+/+^ *N* = 9, *slc9a3r1*^+/-^ *N* = 10, *slc9a3r1*^R180Q/R180Q^ *N* = 6). **(C)** 3D reconstruction of the saccular hair cell layer. brn3c:mGFP signal labels the membrane of the hair cells while red signal labels the kinocilia. **(D)** Hair cell polarization within the saccular macula of *slc9a3r1* KI and their wt siblings.

The reduced capacity of *slc9a3r1* mutant larvae to respond to acoustic stimuli could have several explanations such as: (a) a reduced number or a defective maturation of the sensory hair cells leading to not functional hair cells in the saccular macula, (b) an abnormal morphology or an altered orientation of the sensory hair cells. As regards to (a), 9 WT animals compared to 10 heterozygous mutants and 6 homozygous mutants were tested. No differences in the total number (35.79 ± 6.11 cells for WT, 38.32 ± 5.11 cells for R180Q/R180Q, 43.18 ± 6.53 for ± total cells) or fully mature hair cells (31.16 ± 5.22 cells for WT, 32.26 ± 3.25 cells for R180Q/R180Q, 35.56 ± 3.27 for +/R180Q total cells) among *slc9a3r1*^R180Q/R180Q^*^,^ slc9a3r1*^+/R180Q^ mutants and their Wt siblings were observed (Figure 6A,B). As regards to (b) 6 dpf larvae (*slc9a3r1*^+/+^ *n* = 9, *slc9a3r1*^+/R180Q^ *n* = 10, *slc9a3r1*^R180Q/R180Q^ *n* = 6) were cryosectioned and an immunohistochemistry against the acetylated-tubulin, that labels the kinocilium, was performed. Data analysis revealed no differences in hair cell body morphology or size, hair cell bundle organization or kinocilia/stereocilia length suggesting that the KI do not alter the hair cell morphology (Figure 6C) but induce a reduced response to sound stimuli due to other events. Moreover, no statistically significant differences were observed in the orientation of the hair cells within the sensory patch (see Figure 6D), suggesting that the hearing impairment in KI is not due to hair cell altered polarization. All together these findings indicate the KI hearing impairment is not caused by alterations in the hair cell population.

#### Count of Neurons

Since the hair cells of *slc9a3r1* mutants do not show any defect, the number of neurons in the posterior stato-acoustic ganglion (SAG), which innervate the saccular macula and could be related to a hearing loss, was checked.

**FIGURE 7:**
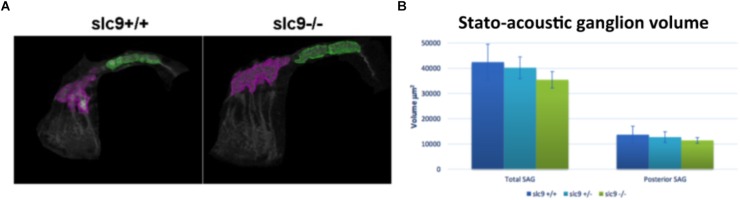
Stato-acoustic ganglion (SAG) characterization. **(A)** 3D reconstruction of the SAG in *slc9a3r1* KI and their WT siblings (number of tested animals: *slc9a3r1*^+/+^ *N* = 3, *slc9a3r1*^+/-^ *N* = 6, *slc9a3r1*^R180Q/R180Q^ *N* = 6). The anterior part of the SAG is depicted in pink, while in green is indicated the posterior part which innervates the saccular macula and it is related to the auditory function. **(B)** The graph shows the total SAG volume (volume of the pink and green area) and the volume of the posterior SAG of *slc9a3r1* animals. Although small differences (not statistically significant) no alterations were observed in the volume of the posterior stato-acoustic ganglion.

In 6 dpf larvae (3 *slc9a3r1^+/+^*, 6 *slc9a3r1*^R180Q/R180Q^, 6 *slc9a3r1*^+/R180Q^ animals used) few differences (although not statistically significant) in the volume of the total SAG (42.439 μm^3^ for *slc9a3r1^+/+^*, 35.454 μm^3^ for *slc9a3r1*^R180Q/R180Q^, 40.237 μm^3^ for *slc9a3r1*^+/R180Q^), and no alterations in the volume of the posterior SAG (13.676 μm^3^ for *slc9a3r1^+/+^*, 11.404 μm^3^ for *slc9a3r1*^R180Q/R180Q^, 12.709 μm^3^ for *slc9a3r1*^+/R180Q^) were detected (Figure 7A,B), thus suggesting that the hearing impairment is not due to a reduction of neurons in this structure.

## Discussion

Despite significant research efforts carried out, the genetics risk factors involved in ARHL are still mainly unknown and only few ARHL susceptibility genes have been detected so far.

Zebrafish mutants can show morphological and functional defects similar to those of other species (Ernest et al., 2000) and their vestibular-acoustic system is very similar to that of human beings, thus representing a cost and time-saving alternative to mouse model allowing a rapid assessment of inner ear function (Baxendale and Whitfield, 2016; Leventea et al., 2016). Furthermore, a number of genes required for hair-cell function in the zebrafish have been already associated with auditory defects in mice and humans, thus revealing their conservation across species (Coimbra et al., 2002).

Using a combination of genetics/genomics studies followed by functional studies we deeply investigated the role of *SLC9A3R1* in ARHL. In particular, the identification of an ultra-rare human mutation [c.539G > A, p.(R180Q)] in two ARHL patients from an isolated community of the Italian Alps led to a series of functional studies including the generation of the first KI zebrafish model for hearing phenotype developed so far demonstrating the pathogenic effect of this variant and thus a possible relevant role of *SLC9A3R1* in ARHL. *SLC9A3R1* encodes the Na^+^/H^+^ exchange regulatory factor 1 (NHERF1) protein (also called Ezrin-radixin-moesin-binding protein of 50 kDa, Ebp50), which belongs to NHERF family of scaffolding proteins. These proteins play key roles in mediating a number of transports and signaling phenomena, interacting with different molecular partners. Interestingly, several protein–protein interactions involving NHERFs take place in the cochlea, and accordingly the Nherf1 KO mouse shows hearing defects, due to hair cells anomalies (Kamiya et al., 2014). NHERF1 interacts with many different molecular partners through its PDZ domains. The missense variant here identified affects the second PDZ domain of the protein (PDZ2) and involves the R180 residue, which is essential for protein–protein interaction, as demonstrated by Mamonova et al. (2012). Accordingly, our protein modeling confirmed this data, suggesting that the p.(R180Q) mutation induces changes in the network of interactions by altering the total number of hydrogen bonds and thus compromising the binding activity of the protein itself. Moreover, the KI zebrafish model further support the pathogenic effect of the p.(R180Q) variant, showing that it causes a reduction in size of the saccular otolith, a structure corresponding to human otoconia whose abnormalities might cause vertigo, dizziness and isolated hearing loss. Zebrafish otoliths are biomineralised structures required for balance and hearing. While the utricular otolith is mainly involved in balancing, the saccular one is implicated in the sense of hearing (Stooke-Vaughan et al., 2015). Interestingly, genetic screenings analyzing several zebrafish mutants with hearing phenotype showed abnormalities in the otolith volume and formation, and revealed that otolith defects are not necessarily associated with other morphological changes of the inner ear (Schibler and Malicki, 2007) as it happens to our *slc9a3r1* KI model. This finding clearly justifies the absence of any balance problem in our KI model and this is in agreement with the ARHL phenotype of the two ARHL patients here described (i.e., high-frequencies hearing loss without any vestibular symptoms). Furthermore, data obtained for *SLC9A3R1* gene are also in agreement with those reported for other genes involved in the otoconia development such as Claudin and Tectorin, in which vestibular signs are frequently absent suggesting a functional compensation by other proteins (Lundberg et al., 2015).

Molecular mechanism underlying KI morphological changes still need to be understood, however, it has been shown that different Na^+^/K^+^-ATPase genes are essential for otolith formation in zebrafish (Blasiole et al., 2006). In this light, a relevant role might be played by the Na^+^/K^+^-ATPase α1 subunit that binds the PDZ2 domain of NHERF1 in opossum kidney cells (Mahon et al., 2003). Since the strong homology of this subunit between zebrafish and opossum sequences (∼71%) it is reasonable to assume that this binding could also happen in zebrafish. Taking into account that our KI model carries a mutation in the PDZ2 domain in a residue critical for protein–protein interaction, it is highly probable that this function could be severely compromised (Voltz et al., 2001; Blasiole et al., 2006) thus leading to an altered otolith formation and a subsequent hearing loss. Furthermore, this mechanism of action could also explain the phenotype’s difference between the KO mouse (that shows anomalies in the shape of the hair cells bundles) and our KI zebrafish model, which specifically reproduces the variant identified in our patients.

Despite carrying the same variant, the KI zebrafish model displays early-onset hearing loss, while the two patients here described are affected by ARHL. This difference might be explained by several hypotheses.

First of all, even though animal models are extremely useful for recapitulating many human diseases, it is important to keep in mind that in some case the phenotypes’ spectra may be different between the two species. Having said that, the *slc9a3r1*^+/R180Q^ model does not show a statistically significant reduction of the hearing thresholds, despite presenting an altered otolith’s volume compared to the WT. In this light, considering that the human patients carry the *SLC9A3R1* variant at the heterozygous state, it is reasonable to think that also in this case, despite the presence of an anatomical defect, the hearing phenotype does not occur at an early age.

## Conclusion

Our results confirmed the role of *SLC9A3R1* in the hearing system function and development, strengthening the previously reported data of the mouse KO model (Kamiya et al., 2014). Moreover, since the KI zebrafish model generated in the present study carries the specific variant identified in two ARHL human patients, this work provides evidence that *SLC9A3R1* gene might play a role in human ARHL. Overall these findings suggest that *SLC9A3R1* could act as a likely Mendelian gene, with a large effect size, in the etiopathogenesis of the late-onset hearing loss form detected in our patients. In this light, larger patients cohorts should be screened at molecular level for the presence of *SLC9A3R1* gene variants/mutations to understand the overall contribution of this gene to the disease itself. Moreover, the identification of other ARHL patients carrying *SLC9A3R1* variants would be also essential for the development of new plans for disease’s prevention and treatment.

## Data Availability

Sequencing data are available at the European Genome-phenome Archive (EGA) at the following link https://ega-archive.org/studies/EGAS00001003072.

## Author Contributions

GG: study design assessment, data production and analysis, and writing the manuscript. AM: data production and analysis and writing the manuscript. NK: protein modeling and molecular dynamics simulation. MC: raw data analysis and quality control. BM: raw data analysis and quality control. SB: *in vitro* studies. MLB: data production. MDS: support of functional studies and conceiving the experiments. PG: conceiving the experiments and writing the manuscript.

## Conflict of Interest Statement

The authors declare that the research was conducted in the absence of any commercial or financial relationships that could be construed as a potential conflict of interest.

## Footnotes

1 https://www.ncbi.nlm.nih.gov/projects/SNP/

2 http://www.1000genomes.org/

3 http://evs.gs.washington.edu

4 http://exac.broadinstitute.org/about

5 http://gnomad.broadinstitute.org/

6 www.pymol.org

7 http://bioinfo.ut.ee/primer3/

## Supplementary Material

FIGURE S1 Clinical features and DNA sequence chromatograms of the two ARHL patients. **(A)** Audiograms of patients 582130 and 5934856; the downward slope indicates that high frequencies are severely affected. **(B)** The figure displays DNA sequence chromatograms showing the nucleotide variant identified in the two ARHL patients and all the details of the mutation. rs ID, Reference SNP ID from dbSNP database. Frequency, frequency of gnomAD Exome all is reported. Predictor tools, GERP++ (higher number is more conserved, >0 is generally conserved), PhyloP (Pathogenicity score: conserved > 0.95, not conserved < 0.95), Polyphen-2 (Pathogenicity score: probably damaging: D, possibly damaging: P, benign: B), SIFT (Pathogenicity score: D: disease causing, N: polymorphism, P: polymorphism automatic), MutationTaster (Pathogenicity score: D:disease causing, N:polymorphism, P: polymorphism automatic), CADD Phred (Pathogenicity score: >10 predicted to be deleterious).

FIGURE S2 *In vitro* experiments: mRNAs quantification and proteins analyses. After cells transfection with expression vectors containing either the WT or the mutant *SLC9A3R1* cDNA, mRNA and protein analyses were performed. **(A)** qRT-PCR on mRNA showed no difference in the expression levels of the mutant (^∗^) compared to the WT. **(B)** Western blot analysis demonstrated that WT and mutated protein are equally expressed. **(C)** Immunolocaliation showed that both the WT and the mutated proteins have a cytoplasmic expression.

## References

[B1] Abbas L. Whitfield T. T. (2009). Nkcc1 (Slc12a2) is required for the regulation of endolymph volume in the otic vesicle and swim bladder volume in the zebrafish larva. *Development* 136 2837–2848. 10.1242/dev.034215 19633174 PMC2730410

[B2] Adzhubei I. Jordan D. M. Sunyaev S. R. (2013). Predicting functional effect of human missense mutations using PolyPhen-2. *Curr. Protoc. Hum. Genet.* 76 7.20.1–7.20.41. 10.1002/0471142905.hg0720s76 23315928 PMC4480630

[B3] Baxendale S. Whitfield T. T. (2016). Methods to study the development, anatomy, and function of the zebrafish inner ear across the life course. *Methods Cell Biol.* 134 165–209. 10.1016/bs.mcb.2016.02.007 27312494

[B4] Berendsen H. J. C. Postma J. P. M. van Gunsteren W. F. DiNola A. Haak J. R. (1984). Molecular dynamics with coupling to an external bath. *J. Chem. Phys.* 81 3684–3690. 10.1063/1.448118

[B5] Berendsen H. J. C. Postma J. P. M. Van Gunsteren W. F. Hermans J. (1981). Interaction models for water in relation to protein hydration. *Intermol. Forces* 14 331–342. 10.1007/978-94-015-7658-1_21

[B6] Bhandiwad A. A. Zeddies D. G. Raible D. W. Rubel E. W. Sisneros J. A. (2013). Auditory sensitivity of larval zebrafish (*Danio rerio*) measured using a behavioral prepulse inhibition assay. *J. Exp. Biol.* 216 3504–3513. 10.1242/jeb.087635 23966590 PMC3749908

[B7] Bhattacharya S. Dai Z. Li J. Baxter S. Callaway D. J. E. Cowburn D. (2010). A conformational switch in the scaffolding protein NHERF1 controls autoinhibition and complex formation. *J. Biol. Chem.* 285 9981–9994. 10.1074/jbc.M109.074005 20042604 PMC2843244

[B8] Blasiole B. Canfield V. A. Vollrath M. A. Huss D. Mohideen M.-A. P. K. Dickman J. D. (2006). Separate Na,K-ATPase genes are required for otolith formation and semicircular canal development in zebrafish. *Dev. Biol.* 294 148–160. 10.1016/J.YDBIO.2006.02.034 16566913

[B9] Bovo R. Ciorba A. Martini A. (2011). Environmental and genetic factors in age-related hearing impairment. *Aging Clin. Exp. Res.* 23 3–10. 10.1007/BF03324947 21499014

[B10] Bowl M. R. Dawson S. J. (2015). The mouse as a model for age-related hearing loss - a mini-review. *Gerontology* 61 149–157. 10.1159/000368399 25471225

[B11] Chun S. Fay J. C. (2009). Identification of deleterious mutations within three human genomes. *Genome Res.* 19 1553–1561. 10.1101/gr.092619.109 19602639 PMC2752137

[B12] Coimbra R. S. Weil D. Brottier P. Blanchard S. Levi M. Hardelin J.-P. (2002). A subtracted cDNA library from the zebrafish (*Danio rerio*) embryonic inner ear. *Genome Res.* 12 1007–1011. 10.1101/gr.227502 12045154 PMC1383735

[B13] Cong L. Ran F. A. Cox D. Lin S. Barretto R. Habib N. (2013). Multiplex genome engineering using CRISPR/Cas systems. *Science* 339 819–823. 10.1126/science.1231143 23287718 PMC3795411

[B14] de Esch C. van der Linde H. Slieker R. Willemsen R. Wolterbeek A. Woutersen R. (2012). Locomotor activity assay in zebrafish larvae: influence of age, strain and ethanol. *Neurotoxicol. Teratol.* 34 425–433. 10.1016/J.NTT.2012.03.002 22484456

[B15] Di Donato V. Auer T. O. Duroure K. Del Bene F. (2013). Characterization of the calcium binding protein family in zebrafish. *PLoS One* 8:e53299. 10.1371/journal.pone.0053299 23341937 PMC3547026

[B16] Ernest S. Rauch G. J. Haffter P. Geisler R. Petit C. Nicolson T. (2000). Mariner is defective in myosin VIIA: a zebrafish model for human hereditary deafness. *Hum. Mol. Genet.* 9 2189–2196. 10.1093/hmg/9.14.2189 10958658

[B17] Friedman R. A. Van Laer L. Huentelman M. J. Sheth S. S. Van Eyken E. Corneveaux J. J. (2009). GRM7 variants confer susceptibility to age-related hearing impairment. *Hum. Mol. Genet.* 18 785–796. 10.1093/hmg/ddn402 19047183 PMC2638831

[B18] Girotto G. Pirastu N. Sorice R. Biino G. Campbell H. d’Adamo A. P. (2011). Hearing function and thresholds: a genome-wide association study in European isolated populations identifies new loci and pathways. *J. Med. Genet.* 48 369–374. 10.1136/jmg.2010.088310 21493956

[B19] Girotto G. Vuckovic D. Buniello A. Lorente-Cánovas B. Lewis M. Gasparini P. (2014). Expression and replication studies to identify new candidate genes involved in normal hearing function. *PLoS One* 9:e85352. 10.1371/journal.pone.0085352 24454846 PMC3891868

[B20] Haddon C. Lewis J. (1996). Early ear development in the embryo of the zebrafish, *Danio rerio*. *J. Comp. Neurol.* 365 113–128. 10.1002/(SICI)1096-9861(19960129)365:1<113::AID-CNE9>3.0.CO;2-6 8821445

[B21] Hess B. Bekker H. Berendsen H. J. C. Fraaije J. G. E. M. (1997). 3 LINCS: a linear constraint solver for molecular simulations. *J. Comput. Chem.* 18 1463–1472. 10.1002/(SICI)1096-987X(199709)18:12<1463::AID-JCC4>3.0.CO;2-H

[B22] Hess B. Kutzner C. Van Der Spoel D. Lindahl E. (2008). GROMACS 4: algorithms for highly efficient, load-balanced, and scalable molecular simulation. *J. Chem. Theory Comput.* 4 435–447. 10.1021/ct700301q 26620784

[B23] Howe K. Clark M. D. Torroja C. F. Torrance J. Berthelot C. Muffato M. (2013). The zebrafish reference genome sequence and its relationship to the human genome. *Nature* 496 498–503. 10.1038/nature12111 23594743 PMC3703927

[B24] Huang Q. Tang J. (2010). Age-related hearing loss or presbycusis. *Eur. Arch. Otorhinolaryngol.* 267 1179–1191. 10.1007/s00405-010-1270-7 20464410

[B25] Inoue M. Tanimoto M. Oda Y. (2013). The role of ear stone size in hair cell acoustic sensory transduction. *Sci. Rep.* 3:2114. 10.1038/srep02114 23817603 PMC3698489

[B26] Kamiya K. Michel V. Giraudet F. Riederer B. Foucher I. Papal S. (2014). An unusually powerful mode of low-frequency sound interference due to defective hair bundles of the auditory outer hair cells. *Proc. Natl. Acad. Sci. U.S.A.* 111 9307–9312. 10.1073/pnas.1405322111 24920589 PMC4078795

[B27] Kidd Iii A. R. Bao J. (2012). Recent advances in the study of age-related hearing loss: a mini-review. *Gerontology* 58 490–496. 10.1159/000338588 22710288 PMC3766364

[B28] Kingston R. E. Chen C. A. Rose J. K. Kingston R. E. Chen C. A. Rose J. K. (2003). Calcium phosphate transfection. *Curr. Protoc. Mol. Biol.* 63 9.1.1–9.1.11. 10.1002/0471142727.mb0901s63 18265332

[B29] Krishnamoorthy N. Yacoub M. H. Yaliraki S. N. (2011). A computational modeling approach for enhancing self-assembly and biofunctionalisation of collagen biomimetic peptides. *Biomaterials* 32 7275–7285. 10.1016/j.biomaterials.2011.06.074 21794910

[B30] Leventea E. Hazime K. Zhao C. Malicki J. (2016). Analysis of cilia structure and function in zebrafish. *Methods Cell Biol.* 133 179–227. 10.1016/bs.mcb.2016.04.016 27263414

[B31] Livak K. J. Schmittgen T. D. (2001). Analysis of relative gene expression data using real-time quantitative PCR and the 2-ΔΔCT method. *Methods* 25 402–408. 10.1006/meth.2001.1262 11846609

[B32] Lundberg Y. W. Xu Y. Thiessen K. D. Kramer K. L. (2015). Mechanisms of otoconia and otolith development. *Dev. Dyn.* 244 239–253. 10.1002/dvdy.24195 25255879 PMC4482761

[B33] Mahon M. J. Cole J. A. Lederer E. D. Segre G. V. (2003). Na + /H + exchanger-regulatory factor 1 mediates inhibition of phosphate transport by parathyroid hormone and second messengers by acting at multiple sites in opossum kidney cells. *Mol. Endocrinol.* 17 2355–2364. 10.1210/me.2003-0043 12881509

[B34] Mamonova T. Kurnikova M. Friedman P. A. (2012). Structural basis for NHERF1 PDZ domain binding. *Biochemistry* 51 3110–3120. 10.1021/bi201213w 22429102 PMC3323774

[B35] Miyamoto S. Kollman P. A. (1992). Settle: an analytical version of the SHAKE and RATTLE algorithm for rigid water models. *J. Comput. Chem.* 13 952–962. 10.1002/jcc.540130805

[B36] Morgan A. Vuckovic D. Krishnamoorthy N. Rubinato E. Ambrosetti U. Castorina P. (2018). Next-generation sequencing identified SPATC1L as a possible candidate gene for both early-onset and age-related hearing loss. *Eur. J. Hum. Genet.* 27 70–79. 10.1038/s41431-018-0229-9 30177775 PMC6303261

[B37] National Health and Nutrition Examination Survey [NHANES] (2015). National Health and Nutrition Examination Survey. Available at: http://www.cdc.gov/nchs/nhanes.htm [accessed September 6, 2015].

[B38] Ng P. C. Henikoff S. (2003). SIFT: predicting amino acid changes that affect protein function. *Nucleic Acids Res.* 31 3812–3814. 10.1093/nar/gkg509 12824425 PMC168916

[B39] Nicolson T. (2005). The genetics of hearing and balance in zebrafish. *Annu. Rev. Genet.* 39 9–22. 10.1146/annurev.genet.39.073003.105049 16285850

[B40] Ohgami N. Iida M. Yajima I. Tamura H. Ohgami K. Kato M. (2013). Hearing impairments caused by genetic and environmental factors. *Environ. Health Prev. Med.* 18 10–15. 10.1007/s12199-012-0300-z 22899349 PMC3541815

[B41] Pittman A. J. Law M.-Y. Chien C.-B. (2008). Pathfinding in a large vertebrate axon tract: isotypic interactions guide retinotectal axons at multiple choice points. *Development* 135 2865–2871. 10.1242/dev.025049 18653554 PMC2562560

[B42] Pollard K. S. Hubisz M. J. Rosenbloom K. R. Siepel A. (2010). Detection of nonneutral substitution rates on mammalian phylogenies. *Genome Res.* 20 110–121. 10.1101/gr.097857.109 19858363 PMC2798823

[B43] Riley B. B. Phillips B. T. (2003). Ringing in the new ear: resolution of cell interactions in otic development. *Dev. Biol.* 261 289–312. 10.1016/S0012-1606(03)00245-8 14499642

[B44] Schibler A. Malicki J. (2007). A screen for genetic defects of the zebrafish ear. *Mech. Dev.* 124 592–604. 10.1016/j.mod.2007.04.005 17574823

[B45] Schindelin J. Arganda-Carreras I. Frise E. Kaynig V. Longair M. Pietzsch T. (2012). Fiji: an open-source platform for biological-image analysis. *Nat. Methods* 9 676–682. 10.1038/nmeth.2019 22743772 PMC3855844

[B46] Schwarz J. M. Rödelsperger C. Schuelke M. Seelow D. (2010). MutationTaster evaluates disease-causing potential of sequence alterations. *Nat. Methods* 7 575–576. 10.1038/nmeth0810-575 20676075

[B47] Stooke-Vaughan G. A. Obholzer N. D. Baxendale S. Megason S. G. Whitfield T. T. (2015). Otolith tethering in the zebrafish otic vesicle requires Otogelin and α-Tectorin. *Development* 142 1137–1145. 10.1242/dev.116632 25758224 PMC4360185

[B48] Takayama K. Igai K. Hagihara Y. Hashimoto R. Hanawa M. Sakuma T. (2017). Highly efficient biallelic genome editing of human ES/iPS cells using a CRISPR/Cas9 or TALEN system. *Nucleic Acids Res.* 45 5198–5207. 10.1093/nar/gkx130 28334759 PMC5435997

[B49] Thisse B. Heyer V. Lux A. Alunni V. Degrave A. Seiliez I. (2004). Spatial and temporal expression of the zebrafish genome by large-scale in situ hybridization screening. *Methods Cell Biol.* 77 505–519. 10.1016/S0091-679X(04)77027-2 15602929

[B50] Van Der Spoel D. Lindahl E. Hess B. Groenhof G. Mark A. E. Berendsen H. J. C. (2005). GROMACS: fast, flexible, and free. *J. Comput. Chem.* 26 1701–1718. 10.1002/jcc.20291 16211538

[B51] van Gunsteren W. F. (1996). *Biomolecular Simulation: The GROMOS 96 Manual Und User Guide*. Zürich: VDF Hochschulverlag AG an der ETH Zürich.

[B52] Van Laer L. Huyghe J. R. Hannula S. Van Eyken E. Stephan D. A. Mäki-Torkko E. (2010). A genome-wide association study for age-related hearing impairment in the Saami. *Eur. J. Hum. Genet.* 18 685–693. 10.1038/ejhg.2009.234 20068591 PMC2987344

[B53] Voltz J. W. Weinman E. J. Shenolikar S. (2001). Expanding the role of NHERF, a PDZ-domain containing protein adapter, to growth regulation. *Oncogene* 20 6309–6314. 10.1038/sj.onc.1204774 11607833

[B54] Vuckovic D. Biino G. Panu F. Pirastu M. Gasparini P. Girotto G. (2013). Lifestyle and normal hearing function in Italy and Central Asia: the potential role of coffee. *Hear. Balanc. Commun.* 11 218–223. 10.3109/21695717.2013.817134

[B55] Vuckovic D. Biino G. Panu F. Pirastu M. Gasparini P. Girotto G. (2014). Age related hearing loss and level of education: an epidemiological study on a large cohort of isolated populations. *Hear. Balanc. Commun.* 12 94–98. 10.3109/21695717.2014.911472

[B56] Vuckovic D. Dawson S. Scheffer D. I. Rantanen T. Morgan A. Di Stazio M. (2015). Genome-wide association analysis on normal hearing function identifies PCDH20 and SLC28A3 as candidates for hearing function and loss. *Hum. Mol. Genet.* 24 5655–5664. 10.1093/hmg/ddv279 26188009 PMC4572074

[B57] Whitfield T. T. Riley B. B. Chiang M.-Y. Phillips B. (2002). Development of the zebrafish inner ear. *Dev. Dyn.* 223 427–458. 10.1002/dvdy.10073 11921334

[B58] Wolber L. E. Girotto G. Buniello A. Vuckovic D. Pirastu N. Lorente-Cánovas B. (2014). Salt-inducible kinase 3, SIK3, is a new gene associated with hearing. *Hum. Mol. Genet.* 23 6407–6418. 10.1093/hmg/ddu346 25060954 PMC4222365

[B59] Xiao T. Roeser T. Staub W. Baier H. (2005). A GFP-based genetic screen reveals mutations that disrupt the architecture of the zebrafish retinotectal projection. *Development* 132 2955–2967. 10.1242/dev.01861 15930106

