# Supplementary material for: Next Generation Sequencing and Animal Models Reveal SLC9A3R1 as a New Gene Involved in Human Age-Related Hearing Loss
Source: Front Genet. 2019 Feb 26;10:142. doi: 10.3389/fgene.2019.00142 (PMC6399162; doi:10.3389/fgene.2019.00142)
Supplement: FIGURE S1 — Clinical features and DNA sequence chromatograms of the two ARHL patients. (A) Audiograms of patients 582130 and 5934856; the downward slope indicates that high frequencies are severely affected. (B) The figure displays DNA sequence chromatograms showing the nucleotide variant identified in the two ARHL patients and all the details of the mutation. rs ID, Reference SNP ID from dbSNP database. Frequency, frequency of gnomAD Exome all is reported. Predictor tools, GERP++ (higher number is more conserved, >0 is generally conserved), PhyloP (Pathogenicity score: conserved > 0.95, not conserved < 0.95), Polyphen-2 (Pathogenicity score: probably damaging: D, possibly damaging: P, benign: B), SIFT (Pathogenicity score: D: disease causing, N: polymorphism, P: polymorphism automatic), MutationTaster (Pathogenicity score: D:disease causing, N:polymorphism, P: polymorphism automatic), CADD Phred (Pathogenicity score: >10 predicted to be deleterious). [file Data_Sheet_1.PDF]

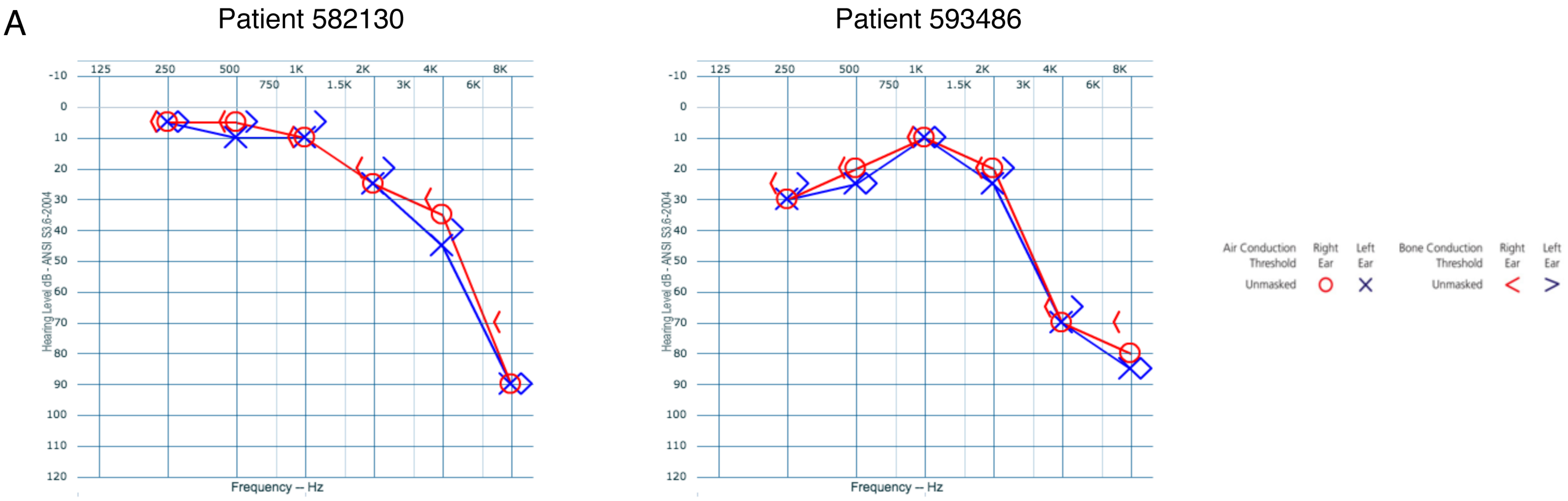

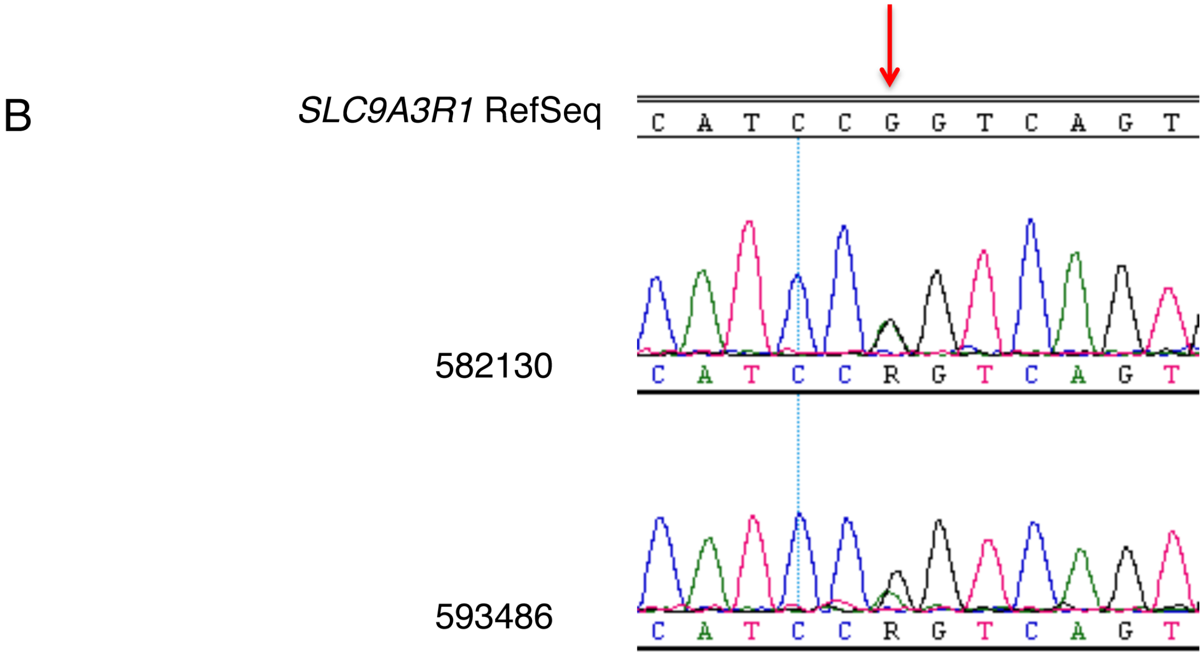

Gene	cDNA change	Amino acid change	rs ID	Frequency (gnomAD)	GERP++	PhyloP	Polyphen-2	SIFT	Mutation Taster	CADD (Phred)
SLC9A3R1	c.539G>A	p.(Arg180Gln)	rs146832150	2,89E-05	3,38	0,997	D	D	D	28,2
