# Supplementary figures and images for: Next Generation Sequencing and Animal Models Reveal SLC9A3R1 as a New Gene Involved in Human Age-Related Hearing Loss
Source: Front Genet. 2019 Feb 26;10:142. doi: 10.3389/fgene.2019.00142 (PMC6399162; doi:10.3389/fgene.2019.00142)

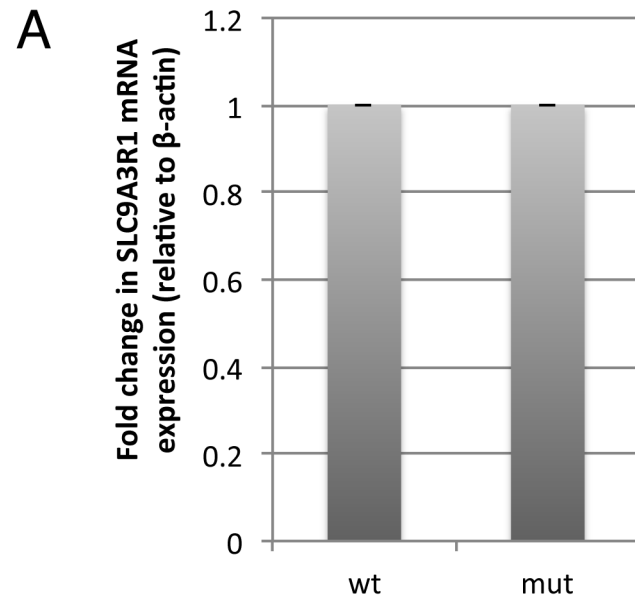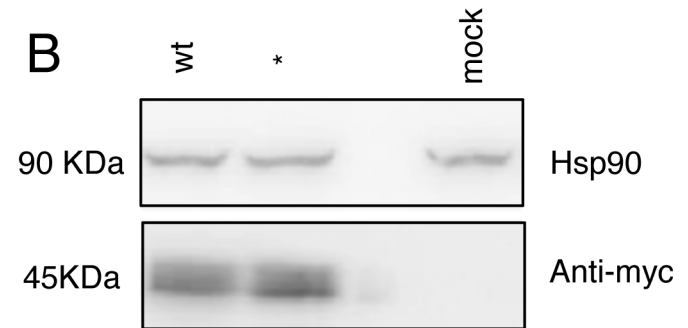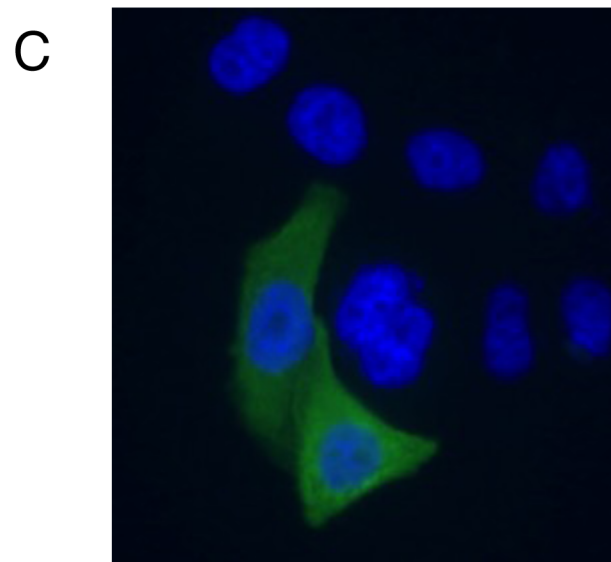

WT

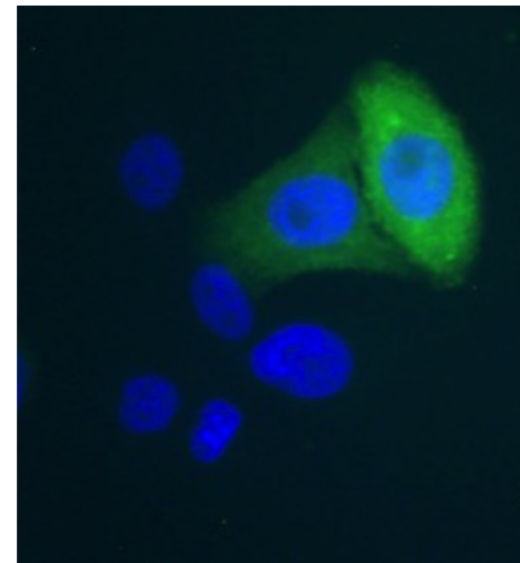

Mut

Supplement: FIGURE S2 — In vitro experiments: mRNAs quantification and proteins analyses. After cells transfection with expression vectors containing either the WT or the mutant SLC9A3R1 cDNA, mRNA and protein analyses were performed. (A) qRT-PCR on mRNA showed no difference in the expression levels of the mutant (∗) compared to the WT. (B) Western blot analysis demonstrated that WT and mutated protein are equally expressed. (C) Immunolocaliation showed that both the WT and the mutated proteins have a cytoplasmic expression. [file Data_Sheet_2.PDF]
